# Brest Cancer Screening—An Alternative View Point

**Published:** 1990-03

**Authors:** Anthony Rodgers

**Affiliations:** Medical Student, Bristol University


### Breast cancer screening—an alternative viewpoint

**Anthony Rodgers**
Medical Student, Bristol University

The purpose of breast cancer screening is to save lives. To have confidence that the national programme can achieve this aim it must be shown firstly that mortality reductions have been conclusively demonstrated in a relevant pilot study and secondly that these benefits can be reproduced in everyday practice.

### EVIDENCE OF MORTALITY REDUCTION

The Forrest report [1] was released when the encouraging results of the Health Insurance Plan (HIP) [2] and Swedish Two Counties Trials [3] were known but since then three randomised trials have failed to show a benefit after screening [4, 5, 6]. The seminal HIP trial suggested mortality reductions but this study is not fully relevant to the UK in 1989. About two-thirds of cancers were detected by clinical examination but the UK programme will rely on mammography alone. The HIP trial employed clinical examination and mammography every year—we shall only be screening triennially. The risk rates of non-attenders in the different populations may also be a reason why the HIP results would overestimate benefits in the UK. The HIP authors concluded that "women with a higher risk of breast cancer tend to self-select themselves for screening" [2], whereas the UK trial experience was the opposite: "women at high risk of breast cancer tend to be non-participants" [4]. It seems that the same screening activity in different countries may produce different results. Another factor to be borne in mind when considering results from the USA is the threshold for biopsy of screen detected lesions. Benign to malignant ratios of 10:1 have been considered acceptable [7], even optimal [8], in America, but the Forrest Report states that a ratio of up to 3:1 should be aimed for [1].

Several case control studies have been performed in Holland [9, 10] and Italy [11] but are unreliable due to the possibility of selection bias, with poor prognosis cases accumulating in the control group. This was illustrated in the UK Trial of Early Detection of Breast Cancer (UKTEDBC) in which 51% of breast cancer deaths in the study group occurred in the 28% of women who did not attend for screening, and the case fatality rate in this group was higher than for control women [4]. Therefore we cannot be sure what fraction, if any, of the lower death rates of attenders in these case control studies was due to the screening process—it may simply have been due to the inherently more favourable risk profiles of women who attend screening.

The Swedish Two Counties, Malmo and UKTEDBC are more relevant and reliable studies. The latest results of the Swedish Two Counties trial suggest 30% fewer breast cancer deaths in the study group (Relative Risk (RR) 0.70, 95% Confidence Interval (CI) 0.55–0.87) [12], but at 9 years follow up the Malmo trial has failed to show any beneficial effect of screening (RR 0.96, 95% CI 0.68–1.35) [5]. The Malmo study has been criticised because of screening in the control group. However a favourable staging distribution was still achieved; there were 19% fewer stage II–IV invasive cancers in the Malmo study group [5], compared to 25% fewer in the Swedish Two Counties study group [3]. Finally, the UKTEDBC failed to show a statistically sound difference between study and control groups, despite the use of annual clinical examination and biennial mammography (RR 0.80, 95% CI 0.64–1.01) [4]. Taken together the Malmo and UKTEDBC trials show the uncertainty of the benefits indicated by earlier studies.

### FROM TRIALS TO EVERYDAY PRACTICE

A successful breast screening programme requires close co-operation between committed radiologists, surgeons, pathologists and community physicians. This was exemplified in the Swedish Two Counties trial but two of the finest centres in the UK at Edinburgh and Guildford failed to reproduce their results. Even if the UKTEDBC results are given the benefit of the statistical doubt they only amount to about 1 death less per 14,000 women screened per year [4]. Whether centres around the country with less experience, resources and motivation can do better seems doubtful. Before the programme over one hundred districts had no mammographic facilities and an equal number had limited experience and obsolete equipment [13] and introduction in less than half of the time recommended by the Royal College of Radiologists may jeopardise whatever benefits were possible [14]. The budget is tight and will not fund many necessary posts [15] and limited resources may be diverted from other clincical activities.

Several other factors may mean that cancer detection is suboptimal. An interval between screening of more than two years [16] and radiologists who are not experts in the field [17] both lead to unacceptable numbers of interval cancers. Work from the Edinburgh breast screening unit suggests that the use of oblique view mammography can allow 11% of cancers to remain undetected and the smallest ones are those most likely to be overlooked [18]. Furthermore we can expect fewer women to attend screening in the UK—the acceptance rates were 91% and 66% in the Swedish Two Counties and UKTEDBC trials respectively. Not only may women in the UK be intrinsically less likely to attend screening but our incomplete population registers may mean that the target population cannot be reached effectivelv [19].

### OTHER BENEFITS OF SCREENING

A negative mammogram may provide reassurance, but the degree of this reassurance should be quantified. Analysis of data from the Guildford arm of the UKTEDBC shows that a clear screen told the woman she was 99.96% sure of not developing breast cancer in the next year—however non-attenders also had a 99.85% chance of being free from the disease in the same time period [20]. Less radical surgery may be possible for some women with cancers detected earlier by screening, but it has proved difficult to quantify any improved psychological readjustment with breast conserving surgery [21].

### DETRACTIONS OF SCREENING

The unsettling effect of screening is likely to be considerable. The Forrest Report estimated that 10% of women will be recalled after their first mammogram, even though only 0.55% will turn out to have cancer. In real terms this will mean that around 380,000 women will be recommended for further assessment after their first mammogram. The title of a recent article "Pensive women, painful vigils—Consequences of delay in the assessment of mammographic abnormalities" [22] sums up the problem. Initial experience at assessment clinics has prompted Baum to write that "we have underestimated the psychological trauma amongst women re-called" [23]. Unfortunately little research has been done in this area but studies of false positive diagnoses in conditions less alarming than cancer have shown worrying results. For example people initially told they were hypertensive but judged normal after three more tests, reported more symptoms of depression and a lower state of general health than a matched group initially found normotensive [24].

False negatives will also occur. 1 in 5 of all cancers in screened women in the UKTEDBC occurred after a negative screen and these women may be particularly devastated when they learn of their diagnosis. False reassurance may also occur in women whose cancers are not missed; unless informed otherwise they may believe that they are certain of a longer life, even cure, if their cancer is detected by screening. However only 1 in 24 women with cancers found by screening in the UKTEDBC benefited in terms of improved survival at 7 years follow up [4]. This means that the majority of women will have longer to live with the knowledge that they have cancer but not longer to live. Furthermore long term follow up of the HIP trial showed that only about one third of all women with prolonged survival had their deaths from breast cancer postponed [25].

Over-diagnosis is a consistent feature of recent breast screening programmes. This was most graphically illustrated in the Malmo trial in which the study group had no improved survival, despite undergoing 145 more operations and living through 966 extra 'breast cancer years'. In the UKTEDBC the rate of detection of carcinoma-in-situ was 5 times greater in the study regions. Post mortem findings suggest that most of these lesions would never have manifested themselves without screening; in a series of consecutive autopsies Nielsen et al found carcinoma-in-situ in 14(18%) of 77 women without previous clinical breast cancer [26]. During the first screening round in the Swedish Two Counties trial demands on inpatient services increased by at least 150% [27], but the Forrest Report makes no allowance for overspill costs to the NHS.

Screening mammography may present considerable medicolegal problems, especially as litigation increases and women have more access to their notes. The situation in the USA should be premonitory. Hall reports that in his experience women will usually insist in having a lesion removed even if the chances of malignancy are 1 in 100 [28]. He quotes a surgical colleague saying "I am biopsying lesions I would not touch in my spouse—the difference is that she will not sue me".

### CONCLUSION

"We embark on breast screening at our peril, with an almost infinite capacity to do harm and a very small window of opportunity for saving lives" [29]. The UKTEDBC and Malmo trials have shown that screening for breast cancer does not reduce mortality from the disease with acceptable consistency. Screening centres around the country cannot reasonably be expected to improve on these results and match the standards reached by the world centres of excellence involved in the Swedish Two Counties study. Every effort must be made to decrease the suffering and death caused by breast cancer in this country but unfortunately the epidemiological evidence suggests that screening will not achieve this. The costs both to well women and the NHS along the way are likely to be considerable—perhaps it is time for a rethink [30].

### REFERENCES

1. FORREST, P. (1986) Breast cancer screening. Report to the Health ministers of England, Wales, Scotland and Northern Ireland. DHSS London HMSO.
2. SHAPIRO, S., STRAX, P., VENET, L. and ROESER, R. (1982) 10–14 year effect of screening on breast cancer mortality. *JNCI*, **69**, 349–355.
3. TABAR, L., GAD, A., HOLMBERG, L. H., LJUNGQUIST, U., EKLUND, G., PETTERSON, F. *et al.* (1985) Reduction in mortality from breast cancer after mass screening with mammography. Randomised trial from the Breast Cancer Screening Working Group of the Swedish National Board of Health and Welfare. *Lancet*, **i**, 829–832.
4. UK Trial of Early Detection of Breast Cancer Group. (1988) First results of mortality reduction in the UK trial of early detection of breast cancer. *Lancet*, **ii**, 411–416.
5. ANDERSSON, I., ASPERGEN, K., JANZON, L., LANDBERG, T., LINDHOLM, K. and LINELL, F. (1988) Mammographic screening and motality from breast cancer: the Malmo mammographic screening trial. *Br Med J*, **297**, 943–950.
6. ROBERTS, M. M., ALEXANDER, F. E., ANDERSON, T. J., CHETTY, U., DONNAN, P. T., FORREST, A. P. M., HEPBOURN, W., HUGGINS, A., KIRKPATRICK, A. E. *et al.* The Edinburgh Trial of screening for Breast Cancer. Mortality results at seven years. Submitted for publication. Lancet 1990, 335: 241–246.
7. BEAHRS, O. H., SHAPIRO, S. and SMART, C. R. (1979) Report of the working group to review the National Cancer Institute—American Cancer Society Breast Cancer Detection Demonstration Projects. JNCI, **62**, 641–709.
8. MOSKOWITZ, M. (1989) Impact of priori medical decisions on screening for breast cancer. *Radiol*, **171**(3), 605–608.
9. VERBEEK, A. L. M., HENDRICKS, J. H. C. L., HOLLAND, R., MRAVUNAC, M., STURMANS, F. and DAY, N. E. (1984) Reduction in mortality from breast cancer through mass screening with modern mammography. First results of the Nijmegen project. *Lancet*, **i**, 1222–1224.
10. COLLETTE, H. J. A., DAY, N. E., ROMBACH, J. J. and DeWAARD. F. (1984) Evaluation of screening for breast cancer in a non randomised study (the DOM project) by means of a case control study. *Lancet*, **i**, 1224–1226.
11. PALLI, D., ROSELLI DEL TURCO, M., BUIATTI, E., CARLI, S., CIATTO, S, TOSCANI, L. *et al.* (1986) A case control study of the efficacy of a non randomised breast cancer screening programme in Florence (Italy). *Int J Cancer*, **38**, 501–504.
12. TABAR, L., FAGERBERG, G., DUFFY, S. W. and DAY, N. E. (1989) The Swedish Two County trial of mammographic screening for breast cancer: recent results and calculation of benefit. *J Epid and Comm Health*, **43**, 107–114.
13. Anonymous. (1987) Screening for breast cancer (Editorial). *Lancet*, **i**, 575–577.
14. WITCOMBE, J. (1988) A license for breast cancer screening? *Br Med J*, **296**, 909–912.
15. WARREN, R. (1988) The debate over mass mammography in Britain; the case for. *Br Med J*, **297**, 969–971.
16. TABAR, L., FAGERBERG, G., DAY, N. E. and HOLMBERG, L. (1987) What is the optimum interval between mammographic screening examinations? *Br J Cancer*, **55**, 547–551.
17. ANDERSSON, I. (1984) What can we learn from interval cancers? *Rec Res Ca Res*, **90**, 161–163.
18. MUIR, B. B., KIRKPATRICK, A. E., ROBERTS, M. M. and DUFFY, S. W. (1984) Oblique-View Mammography: adequacy for screening. Work in progress. *Radiol*, **151**(1), 39–41.
19. BOWLING, A. and JACOBSON, B. (1989) Screening; the inadequacy of population registers. *Br Med J*, **298**, 545–546.
20. THOMAS, B. A., PRICE, L. J., BOULTER, P. S. and GIBBS, N. M. (1984) The first three years of the Guildford breast screening project. *Rec Res Ca Res*, **90**, 195–199.
21. FALLOWFIELD, L. F., BAUM, M. and MAGUIRE, G. P. (1986) Aspects of breast conservation on psychological morbidity associated with diagnosis and treatment of early breast cancer. *Br Med J*, **293**, 1331–1334.
22. FENTIMAN, S. (1988) Pensive women, painful vigils: consequences of delay in assessment of mammographic abnormalities. *Lancet*, **i**, 1041–1042.
23. BAUM, M. Screening in the 1990's—a time for reappraisal. Paper presented at Symposium Mammographicum, 1989.
24. BLOOM, J. R. and MONTERESSA, S. (1981) Hypertension labelling and sense of well-being. *Am J Public Health*, **71**, 1228–1232.
25. SHAPIRO, S., VENET, W., STRAX, P. and VENET, L. (1988) Current results of the breast cancer screening randomised trial: The Health Insurance Plan (HIP) of Greater New York Study. In "Screening for Breast Cancer", pp 3–15. Eds Day, N. E., MILLER, A. B. Hans Huber Publishing, Toronto, Canada.
26. NIELSEN, M., JENSEN, J. and ANDERSEN, J. (1984) Precancerous and cancerous breast lesions during lifetime and at autopsy. *Cancer*, **54**, 612–615.
27. HOLMBERG, D. M., ADAMI, H-O., PERSON, I., LUNDSTROM, T. and TABAR, L. (1986) Demands on surgical inpatient services after mass mammographic screening. *Br Med J*, **293**, 779–782.
28. HALL, F. M. (1986) Screening mammography—potential problems on the horizon. *N Engl J Med*, **314**, 53–55.
29. BAUM, M. (1988) Delayed assessment of mammographic abnormalities (letter). *The Lancet*, **i**, 1218.
30. ROBERTS, M. M. (1989) Breast screening: time for a rethink? *Br Med J*, **299**, 1153–1115.

